# The Role of Adenosine in Schizophrenia: A Literature Review and Perspective

**DOI:** 10.1192/j.eurpsy.2025.2249

**Published:** 2025-08-26

**Authors:** M. Pjevac

**Affiliations:** University Psychiatric Clinic Ljubljana, Ljubljana, Slovenia

## Abstract

**Introduction:**

Schizophrenia is a severe, chronic mental disorder, with about one-third of patients failing to achieve remission. Its multifactorial etiology includes a strong genetic component that remains insufficiently understood. Dysregulation of dopaminergic and glutamatergic neurotransmitter pathways has been implicated in the development of schizophrenia, as revealed through neuromorphological, molecular, pharmacological, and pharmacogenetic studies. Recent research has expanded this focus to include neuroinflammatory mechanisms and metabolic pathways, emphasizing the fine regulation of these neurotransmitter systems. Among these, adenosine has gained attention for its potential role in the pathophysiology of schizophrenia.

**Objectives:**

This literature review aims to explore the role of adenosine in schizophrenia, its connection to dopaminergic and glutamatergic hypotheses, and broader implications for the development of symptoms and complications.

**Methods:**

A comprehensive review of studies investigating adenosine’s neuroregulatory, inflammatory, and neuromodulatory functions.

**Results:**

The adenosine hypothesis suggests that reduced adenosine activity plays a critical role in the development of schizophrenia symptoms. Adenosine regulates dopaminergic and glutamatergic neurotransmission via ADORA A1 and ADORA A2 receptors. Studies have shown changes in adenosine receptors, transporters, and enzymes involved in adenosine metabolism in individuals with schizophrenia. Moreover, adenosine’s neuroprotective role, particularly in stress and inflammation, connects it to the dopaminergic/glutamatergic systems and the broader neurodevelopmental “two-hit” hypothesis, suggesting that schizophrenia develops through a combination of genetic vulnerability and environmental factors. A study by Ary Gadelha and colleagues proposed a link between hypo-adenosine states and the increased risk of sudden cardiac death in schizophrenia patients, indicating the broader systemic importance of adenosine dysregulation in these individuals.

**Conclusions:**

Adenosine appears to be a potential modulator in the pathophysiology of schizophrenia, connecting various neurotransmitter pathways and influencing genetic vulnerabilities, neuroinflammation, and cardiovascular risks. Further research into adenosine-related mechanisms could offer valuable insights into early interventions, improving treatment outcomes and potentially reducing complications such as treatment resistance and sudden cardiac death in schizophrenia patients.

**References**

Lara DR, Souza DO. Schizophrenia: a purinergic hypothesis. Med Hypotheses. 2000;54(2):157-166. doi:10.1054/mehy.1999.0003

Boison D. Adenosine as a neuromodulator in neurological diseases. Curr Opin Pharmacol. 2008;8(1):2-7. doi:10.1016/j.coph.2007.09.002

Fredholm BB, Chen JF, Cunha RA, Svenningsson P, Vaugeois JM. Adenosine and brain function. Int Rev Neurobiol. 2005;63:191-270. doi:10.1016/S0074-7742(05)63007-3. PMID: 15797469

**Disclosure of Interest:**

None Declared

